# Determination of murine norovirus aerosol concentration during toilet flushing

**DOI:** 10.1038/s41598-021-02938-0

**Published:** 2021-12-07

**Authors:** Corey Boles, Grant Brown, Matthew Nonnenmann

**Affiliations:** 1 Cardno ChemRisk, Cincinnati, OH USA; 2 College of Public Health, University of Iowa, Iowa City, IA USA

**Keywords:** Infectious diseases, Applied microbiology

## Abstract

Murine norovirus (MNV) was used as a surrogate for human viral pathogens (*e.g.,* norovirus) to determine if toilet flushing resulted in the aerosolization of virus. A flushometer type toilet was seeded with a viral solution of 10^5^ and 10^6^ PFU mL^-1^ of MNV and then flushed. Upon flushing, two bioaerosol samplers were activated to collect aerosolized MNV. Prior to the experiment, two optical particle counters monitored particle size and number distribution of aerosol produced from flushing a toilet across height, position, and side. The location with the highest mean particle concentration, was behind the toilet and 0.15 m above the toilet bowl rim, which is where bioaerosol sampling occurred. Bioaerosol and toilet water samples were collected, extracted and then quantified using RT-ddPCR. The concentration of MNV collected after seeding the toilet water ranged from 2.18 × 10^5^ to 9.65 × 10^6^ total copies of MNV. Positive samples of airborne MNV were detected with collected concentrations ranging from 383 to 684 RNA copies/m^3^ of air. This study provides evidence that viral pathogens may be aerosolized when a toilet is flushed. Furthermore, the MNV used in this study is a model organism for human norovirus and may be generalizable to other viral pathogens (e.g., coronavirus). This study suggests that virus is aerosolized from toilet flushing and may contribute to human exposure to viral pathogens.

## Introduction

The role of fecal–oral route of human exposure to viral pathogens is well known^1^, however, little is known about the impact of fecal aerosols generated from toilets during the flushing of human waste on the transmission of pathogens. Characterizing and controlling exposure to these aerosols may have significant implications for reducing transmission of viral pathogens such as human norovirus, rotavirus, and Severe Acute Respiratory Syndrome Coronavirus 2 (SARS-CoV-2)^2^.

Globally, NV is the leading cause of sporadic cases and outbreaks of AGE. Norovirus is a significant public health problem. Acute gastroenteritis causes the second greatest burden among infectious diseases globally^3^. Annually in the United States (U.S.), norovirus causes between 19 and 21 million cases of AGE, 1.7–1.9 million outpatient visits, 400,000 emergency department visits, 56,000–71,000 hospitalizations, and 570–800 deaths^4^.

Norovirus is highly contagious and easily transmitted due to several factors. First, the infectious dose is considered low, approximately 18–1000 viral particles^5^. Second, the virus is able to withstand a wide range of temperatures (*i.e.*, 0–60 °C), multiple disinfectants, and survive in water and on environmental surfaces^6–8^. Lastly, NV can be transmitted either directly or indirectly. Sources of transmission include person-to-person contact, environmental contamination, fomites, or droplets containing viral particles^9–12^.

The primary route of transmission of NV is through the fecal–oral route^13^. However, there have been outbreaks where person-to-person contact, and the fecal–oral route have been unable to explain the spread of NV. For example, Gellert et al. was unable to find direct evidence of fecal contact and suggested that NV transmission was airborne^14^. In fact, there is recent evidence that demonstrates that NV can be aerosolized; however, that evidence is limited, and an aerosolization source has yet to be determined.

Information about human exposure to aerosolized NV is lacking. This lack of information is largely due to methodological and logistical challenges. Specifically, air sampling and quantification methods for NV are currently underdeveloped. Therefore, there is a need for simple, easily deployed air sampling methods that can be used to evaluate NV occupational and environmental contamination^15^. Additionally, there are logistical challenges of identifying acute outbreaks in time to conduct aerosol sampling^16^. However, even with the logistical challenges, four studies have detected aerosolized NV in the following settings: wastewater treatment, health care, and during biosolid land application^17–20^. Inferences about aerosol generation sources and airborne NV concentration from these studies are difficult due to the limited locations where sampling took place, and differences in the approach used to sample for aerosolized NV. Nevertheless, these studies provide direct evidence that NV can become aerosolized in hospitals and wastewater treatment facilities.

A common practice in hospitals is to dispose of bowel and vomitus waste from patients infected with NV by toilet flushing^21^. This practice, along with individuals infected with NV using residential bathrooms and NVs resilience in the environment, might explain why NV was detected at a wastewater treatment facility^18,19^. The mechanisms involved when a toilet is flushed have been shown to create aerosols from inside the toilet bowl^22^. Furthermore, two additional studies have found that biological materials (*e.g.*, bacteria and virus) contained in toilet bowl water can also become aerosolized during flushing^23,24^. Barker and Jones (2005) experimentally contaminated toilet bowls (*i.e.,* seeded the toilets) with microorganisms and then flushed the toilet. They successfully detected microorganisms in the air after flushing a seeded toilet^23^. Another study analyzed whether the type of toilet had an impact on aerosol generation, and found that flushometer (FOM) type toilets commonly found in healthcare, educational, and governmental buildings significantly increased the amount of aerosol generated compared to gravity-fed toilets^22^. This study hypothesized that toilet plume airborne infection risks are likely from viruses, of which the most significant is NV^22^. Healthcare workers (HCW) may not currently use any precautions to prevent exposure to aerosolized NV (*i.e.*, workers are at risk as they do not use respiratory protection or face shields when toilet flushing). Therefore, with evidence that flushing toilets can generate bioaerosols, there is a need to evaluate toilet flushing as an aerosolization and exposure source of NV to HCW, patients in a health care setting, and the public.

The aim of this study was to determine if toilet flushing is an aerosol generation source of NV, using a surrogate murine norovirus (MNV). Murine norovirus was selected as a surrogate due to its structural similarities to NV, with MNV being 28 to 35 nm in diameter and icosahedral in shape which is aligned with NV^25–27^. The use of MNV as a surrogate for NV is considered a model surrogate due to similar survival and inactivation responses^25^. Furthermore, MNV has been used as a surrogate for NV in several studies due to the difficulty surrounding the culturability of NV^25^. Therefore, MNV is considered an ideal surrogate for NV compared with more commonly used surrogates such as phages, tracers, Tulane virus, porcine enteric calicivirus, and feline calicivirus^25,27^. To achieve this aim, two objectives were designed to (1) characterize the particle plume produced from the flushing of a FOM type toilet to inform bioaerosol sampler placement and (2) quantify the concentration of aerosolized MNV from flushing a FOM type toilet using two different bioaerosol samplers.

## Materials and methods

### Toilet plume characterization

**Figure 1:**
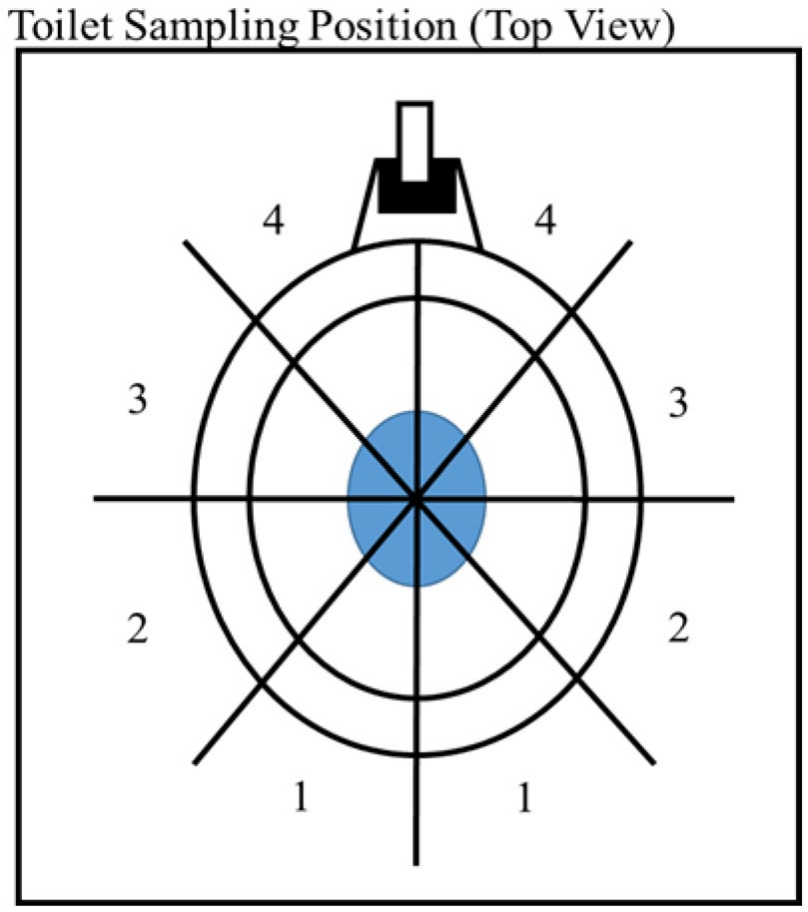
Sampling positions for the PCs to characterize toilet plumes produced from flushing FOM type toilets.

Experiments were conducted using a FOM type toilet, located at The University of Iowa (UI) Research Park in Coralville, IA. The aerosol plume of the FOM type toilet was investigated to inform bioaerosol sampling location around a toilet seeded with murine norovirus (MNV). The toilet plume droplet size and count distribution were measured using a TSI AeroTrak Particle Counter (OPC, TSI, Shoreview, MN, USA). Two OPC instruments were used simultaneously at opposite locations around the toilet bowl. The OPCs were activated for five minutes prior to toilet flushing to determine background particle concentrations and size distribution. After five minutes, the toilet was flushed. The OPC instruments were operated for an additional 30 min after flushing. After sampling the OPCs were moved to another location until all positions were sampled (Fig. 1). The OPCs were placed 0.15 m away from the toilet for each trial. Three heights were sampled for each location: level with toilet bowl, 0.15 m above toilet bowl, and 0.25 m above toilet bowl. Three trials were performed for each position and height for a total of 72 measurements. The position and height with the highest arithmetic mean particle concentrations were used for bioaerosol sampling.

### Toilet seeding

The inside of the toilet (containing 3.1 L of water), outside of the toilet, and the floor around the toilet were cleaned using a 1:128 dilution of Virex Plus (Diversey, Charlotte, NC, USA) following manufactures recommendations for norovirus.

Murine norovirus (Dr. Skip Virgin’s Laboratory, Washington University, St. Louis, MO) was used as a human norovirus surrogate for this project. Stock concentrations of MNV were 10^7^ plaque forming units (PFU)/mL stored in Dulbecco’s Modified Eagle Medium (DMEM)^27^.

The FOM toilet bowl was seeded with 50 mL of MNV solution diluted to concentrations of 10^5^ and 10^6^ PFU mL^-1^, which is a concentration similar to NV detected in vomit and feces from infected people^28–30^. The viral solution was diluted with Hanks Balanced Salt Solution (HBSS). The sidewalls of the toilet bowl were coated with 25 mL of MNV viral solution, and the remaining 25 mL was stirred into the 3.1 L of toilet bowl water.

### Bioaerosol sampling

**Figure 2:**
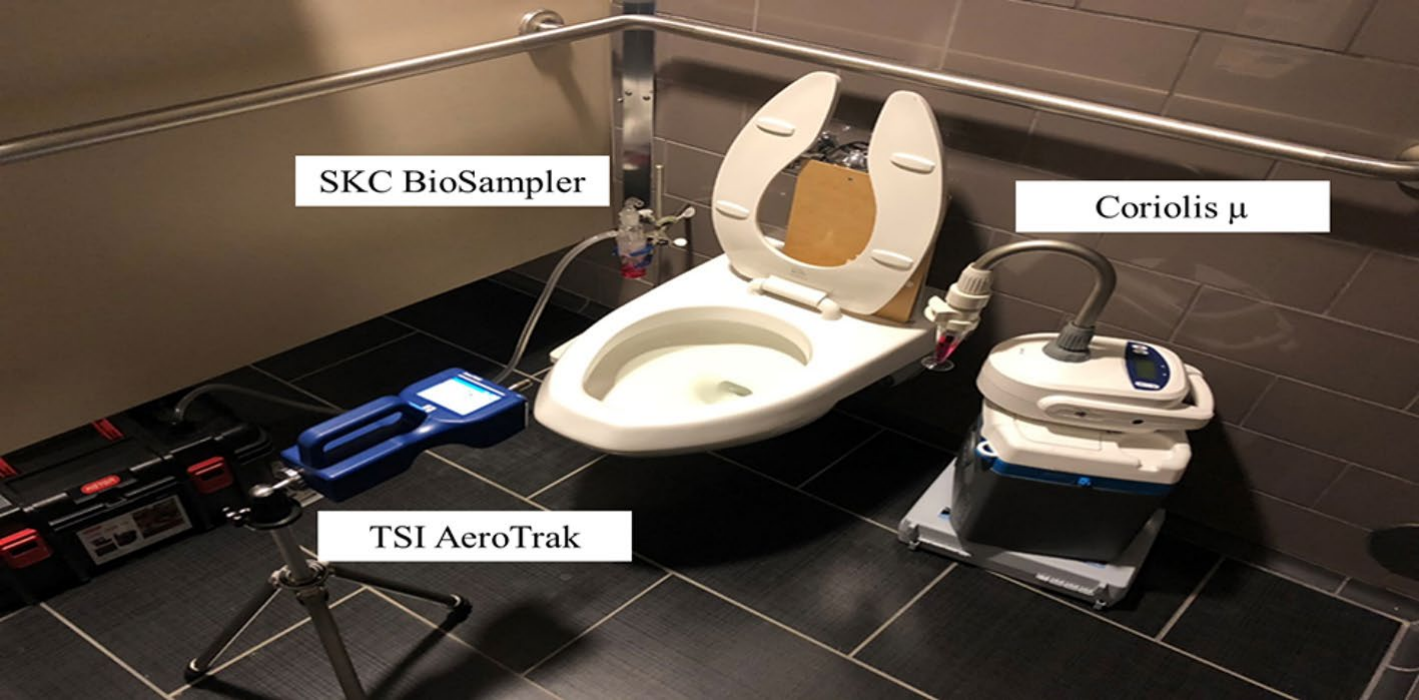
Sampling setup for MNV aerosolization experiments around an FOM toilet located at ITF on UI Research Park.

Aerosol samples were collected using a SKC BioSampler (SKC Inc., Eighty Four, PA, USA) and a Coriolis µ sampler (Bertin Technologies, France). Samplers were placed at a priori determined sampling positions based on the highest concentration of particles generated during toilet flushing. The SKC BioSampler and the Coriolis µ were operated at 12.5 and 150 L per minute (l min^-1^), respectively. Furthermore, the SKC BioSampler and the Coriolis µ contained 20 mL and 15 mL of HBSS, respectively. Lastly, an OPC was located 0.15 m from the toilet bowl and measured particle size and count during sampling (Fig. 2).

Prior to experimentation, quality control samples were collected (*i.e.*, bioaerosol and toilet water) to determine if any NV was present prior to our experiment. After toilet seeding, the OPC was activated for 35 min. After 5 min of sampling, the toilet was flushed, and the aerosol samplers were activated for a 30-min sampling periods. Six trials consisting of 12 control samples, 12 aerosol samples, and 6 toilet water samples for a total of 30 samples were conducted. After sampling, the sample was transferred to sterile 50 mL conical tubes and refrigerated. Pre and post calibration of airflow was performed using a primary airflow standard (BGI tetraCal, Mesa Labs, Butler, NJ, USA). Additional information was collected such as room and toilet stall dimensions, relative humidity (RH) and temperature (Hygro-Thermometer, Extech Instruments, Waltham, MA, USA), ventilation airflow (TSI Balometer, Shoreview, MN, USA) and both total and free chlorine, and pH of the toilet bowl (Lovibond MD100 Colorimeter, Tintometer Group, Amesbury, UK).

Samples were transferred to Spin-X UF centrifugal concentrators (Corning, Corning, NY, USA). Samples were spun in a centrifuge at 20 °C for 20 min. After centrifugation, the samples were transferred to a microcentrifuge tube, were brought to final volume of 300 µL using HBSS media and stored at − 80 °C for further analysis. A similar approach has been used previously^17^.

### Viral quantification

Viral RNA was extracted using the QIAamp Viral RNA Mini Kit (Qiagen, Hilden, Germany). RNA was converted to complimentary DNA (cDNA) using the One-Step RT-ddPCR Advanced Kit for Probes (Bio-Rad, Hercules, CA, USA) using 5 µL of isolated RNA in a total reaction volume of 40 µL per instructions. The following primers and probe were used to complete RT-ddPCR of MNV aerosol samples^31^.

- MNV Forward: 5’-GCC CTT GTA CCA CCC TAT TT-3’
- MNV Reverse: 5’-CTC GAC GCA CAT CAA GAA GA-3’
- MNV Probe: 5’-56-FAM - CGC TTT GGA ACA ATG-MGBNFQ

The PCR reactions using primers (IDT Coralville, IA, USA) and probes (Thermo Fisher Waltham, MA, USA) were performed on a Bio-Rad QX200 Droplet Digital PCR System using droplet generation oil for probes (Bio-Rad, Hercules, CA, USA). Reverse Transcription-ddPCR were performed under the following cycling conditions: 1 cycle of 50 °C for 60 min, 1 cycle of 95 °C for 10 min, 40 cycles of 95 °C for 30 s and 60 °C for 1 min, and 1 cycle of 98 °C for 10 min. The ramp rate was set to 2 °C/second.

### Data analysis

Mean particle concentrations for minute six of the toilet plume data (the first minute post flush) were analyzed for particles 0.3, 0.5, 1, 3, 5, 10 µm. The Height and Position with the highest mean particle concentrations across Trial and Side for all particle sizes were selected for sampling locations. Particle concentrations across sampling heights, positions, and side of toilet were compared in R^32^ using the Wilcoxon Signed Rank Test at a type 1 error rate of 0.05.

The ddPCR provides absolute quantification of MNV, a minimum threshold of 10,000 sample droplets were analyzed with a limit of detection being three or more positive droplets.

## Results

### Toilet plume characterization

The height with the highest mean particle concentrations was at 0.15 m above the toilet bowl (Table 1). At a height of 0.15 m, mean particles concentrations were highest for 0.3 µm particles and lowest for 10 um particles. Therefore, a height of 0.15 m was selected for sampler placement. The position with the highest mean particle concentration at a height of 0.15 m varied by particle diameter corresponding to each OPC channel. Mean particle concentration was highest at Position 4 for 0.3 and 0.5 µm particles, Position 3 for 1 µm particles, and Position 2 for 3, 5, and 10 µm particles (Table 2). However, there were no significant differences in mean particle concentration for 1, 3, 5, and 10 µm diameter particles between any positions across all heights (Table 3). Based on these results, it was determined to perform sampling at Position 4, 0.15 m above the toilet. There were significant differences between the left and right sides of the toilet for particle sizes 0.3 and 0.5 µm (p-value = 0.002 for both particle sizes) (Table 4).

**Table 1:** Arithmetic mean (SD) particle count detected across all positions by height and particle size.

Height (m)	Particle size (µm)					
	0.3	0.5	1	3	5	10
0	4.61E+06	2.55E+05	7.88E+04	1.95E+04	1.07E+04	3.25E+03
	(1.41E+06)	(1.28E+05)	(5.84E+04)	(1.33E+04)	(6.62E+03)	(1.85E+03)
0.15	6.51E+06	7.89E+05	2.34E+05	4.03E+04	1.93E+04	4.70E+03
	(3.80E+06)	(3.85E+05)	(1.40E+05)	(3.97E+04)	(2.02E+04)	(3.32E+03)
0.25	5.14E+06	6.51E+05	1.47E+05	1.95E+04	1.05E+04	3.65E+03
	(2.92E+06)	(2.19E+05)	(7.50E+04)	(8.68E+03)	(5.53E+03)	(2.05E+03)

**Table 2:** Arithmetic mean (SD) particle count detected at Height 0.15 m across all positions and particle size.

Position	Particle size (µm)					
	0.3	0.5	1	3	5	10
1	7.19E+06	5.52E+05	1.93E+05	3.96E+04	1.81E+04	3.95E+03
	(3.48E+06)	(1.70E+05)	(1.72E+05)	(4.77E+04)	(2.13E+04)	(3.65E+03)
2	2.69E+06	5.48E+05	2.43E+05	5.77E+04	3.07E+04	7.01E+03
	(4.31E+05)	(2.48E+05)	(1.68E+05)	(5.26E+04)	(2.90E+04)	(3.86E+03)
3	4.85E+06	7.76E+05	2.52E+05	4.08E+04	1.76E+04	4.00E+03
	(4.31E+05)	(2.22E+06)	(3.62E+05)	(1.45E+05)	(3.72E+04)	(1.76E+04)
4	1.13E+07	1.28E+06	2.46E+05	2.33E+04	1.08E+04	3.83E+03
	(6.23E+05)	(1.86E+05)	(9.36E+04)	(1.12E+04)	(4.14E+03)	(2.67E+03)

**Table 3:** *P* values for comparisons between Position across all Heights.

Position comparison	Particle size (µm)					
	0.3	0.5	1	3	5	10
1 versus 2	0.0003*	0.012*	0.6095	0.5509	0.6782	0.8229
1 versus 3	0.0028*	0.5509	0.3927	0.5246	0.1009	0.0392
1 versus 4	0.7660	0.1187	0.2121	0.2081	0.0553	0.0458
2 versus 3	0.3692	0.2462	0.2645	0.9725	0.2895	0.0462
2 versus 4	0.1297	0.004*	0.1964	0.6320	0.3465	0.2078
3 versus 4	0.0023*	0.0182	0.6019	0.8900	0.9661	0.5014

* = statistical significance with a 0.05 threshold.

**Table 4:** *P* values for comparisons between left versus right side of toilet at Height 0.15 m and Position 4, n = 10.

Comparison	Particle size (µm)					
	0.3	0.5	1	3	5	10
Left versus Right	0.002*	0.002*	0.8457	0.5566	1.0000	0.4961

*= statistical significance with a 0.05 threshold.

### Bioaerosol sampling

The location with the highest average particle concentrations was at Position 4, 0.15 m above the toilet bowl. The particle concentration was significantly higher for two particle sizes on the left side of the toilet. Therefore, to optimize the sampling approach, the SKC BioSampler was placed at Position 4 to maximize the likelihood of detection. The Coriolis µ, was placed on the right side of the toilet.

MNV was not detected in aerosol samples collected with both samplers across all three trials for seeding concentration of 10^5^ PFU mL^-1^. The seeded toilet water samples were all positive with a concentration range of 2.18 × 10^5^–6.40 × 10^5^ total RNA copies (Table 5).

**Table 5:** Concentrations of MNV from all trials for control, toilet, and aerosol samples.

Trial n = 30	Seeding concentration (PFU mL^-1^)	Control		Samples		
		Toilet	Air	Toilet (total copies)	SKC BioSampler	Coriolis µ (copies/m^3^)
1	10^5^	ND	ND	6.40 × 10^5^	ND	ND
2	10^5^	ND	ND	2.18 × 10^5^	ND	ND
3	10^5^	ND	ND	3.43 × 10^5^	ND	ND
4	10^6^	ND	ND	9.35 × 10^6^	ND	684
5	10^6^	ND	ND	4.76 × 10^6^	ND	505
6	10^6^	ND	ND	9.65 × 10^6^	ND	383

For a seeding concentration of 10^6^ PFU mL^-1^, aerosol samples collected using the SKC BioSampler were negative [*i.e.*, below the limit of detection (LOD)] across all three trials. Aerosol samples collected using Coriolis µ sampler were positive (*i.e.*, above the LOD) for MNV across all three trials. The concentration of MNV collected using the Coriolis µ sampler ranged 383–684 RNA copies/m^3^. The seeded toilet samples were all positive with a concentration range of 4.76 × 10^6^–9.65 × 10^6^ total RNA copies. All pre-seeding toilet water and aerosol control samples were negative for MNV (Table 5).

The RH ranged from 52.8 to 54.8% with an arithmetic mean of 53.9%. The temperature across all six trials was 22.7–23.4 °C with an arithmetic mean of 23.0 °C. Free and total chlorine from the pre-seeded toilet bowl water was 0.02–0.08 mg l^-1^ and 0.02–0.06 mg l^-1^, respectively. The arithmetic mean concentration for free and total chlorine was 0.05 mg l^-1^ and 0.04 mg l^-1^ across all six trials, respectively. Lastly, the pH from the pre-seeded toilet bowl water ranged from 7.27 to 7.43, and the arithmetic mean pH was 7.36 across all six trials.

The arithmetic mean flow rate for the total return air was 131 cubic feet per minute (CFM) and 180 CFM for the supply air resulting in approximately nine room air changes per hour. Ventilation airflow in the bathroom consisted of two return airflow vents and one supply vent. The supply vent and one return vent were located near the bathroom entrance, and the second return vent was located outside of the toilet stall above the urinal.

## Discussion

In this study, we detected aerosolized MNV after flushing a FOM toilet. The Coriolis µ sampler collected aerosolized MNV in concentrations ranging from 383–684 RNA copies/m^3^. Aerosolized MNV was not detected when using the SKC BioSampler. The Coriolis µ samples air at a higher rate than the SKC BioSampler which may explain why the Coriolis µ was able to detect aerosolized MNV with a toilet seeding concentration of 10^6^ PFU mL^-1^. The sampling approach used in this study was similar to a previous study which used the Coriolis µ sampler in healthcare settings^17^. In addition, the concentrations of aerosolized MNV detected in this study were similar to the concentration of aerosolized NV detected by Bonifait et al. (2015), ranging 13.5–2350 genomes (m^3^)^-1^. With similar detected concentrations across both studies, as well as, similar sampling approaches, it is possible that an aerosolization source of NV detected by Bonifait et al. (2015) was a flushing toilet.

The concentration of RNA copies collected from seeded toilet water (2.18 × 10^5^–9.65 × 10^6^ total copies) were similar to previous studies that examined concentrations of NV in feces and vomit^28,30^. The results observed in this study may be an underestimate as airborne NV virus concentration could toilet water concentration could be as high as 3.2 × 10^11^ virus per mL of bowl water. This estimate is based on the volume of water in the toilet bowl (*i.e.*, 3.1 L), the reported density of human waste (*i.e.*, 1.06 g per ml) (Brown et al., 1996), the mass of waste delivered to the bowl during defecation (*i.e.*, 107 g) (Rose et al., 2015) and assuming perfect mixing in the bowl during flushing. Therefore, flushing a FOM toilet with NV contaminated waste may result in exposure to aerosolized NV among workers, patients, and the public, however, more data and evaluation are required.

There is concern surrounding the risk of infection from inhalation of aerosolized NV and other viral pathogens (*e.g.*, rotavirus, SARS-CoV-2) in various indoor settings (*e.g.,* hospital, restaurant, and long-term care facilities)^11,33–35^. This study is the first to identify a generation source for aerosolized MNV indoors, an NV surrogate. Specifically, the NV capsid is formed from 90 dimers of the major structural protein which evidence suggests is a dynamic structure adapting to in vivo and environmental conditions to maintain infectivity^36^. The resilience of the viral capsid may impact extraction efficiencies compared to other viral surrogates commonly used (*e.g*., MS2 phage). Therefore, experimental observations from environmental studies using MNV as a surrogate for NV are likely more generalizable compared to studies using other viral surrogates (e.g., MS2 phage). Also, the observations of this study may be representative of other aerosolized viral pathogens (e.g., SARS-CoV-2) generated during toilet flushing, however additional data collection is needed. This study provides evidence supporting the hypothesis that toilet flushing is a possible source for aerosolized NV, and potentially other viral pathogens (e.g., SARS-CoV-2)^17,22,35,37^. Only one previous study has determined a generation source for aerosolized NV (*i.e.*, outdoors during the application of biosolids)^20^. Another study was able to detect NV at an air exhaust site of a hospital water-treatment plant (WWTP); however, the specific generation source was not identified^19^. The concentration of aerosolized MNV observed in this study was similar to another study conducted near patients in healthcare facilities^17^. In patient care settings where toilets are used to dispose of human waste, flushing could be a source of aerosolization for NV, which would place the public and healthcare staff at risk for virus exposure. However, care providers may not always use the toilet to dispose of human waste, therefore other routes of exposure are likely more common (*e.g.*, fomite).

Caretakers of individuals infected with NV and SARS-CoV-2 may be exposed to the virus through toilet flushing. The bioaerosol generated from flushing a FOM toilet containing NV may contain up to 680 RNA copies/m^3^. With humans breathing approximately 6 L of air per minute, during a 5-min time period in the bathroom, inhalation exposure would be approximately 20 copies of NV. The reported infectious dose is approximately 18 NV particles, therefore, 5-min exposure in the bathroom could result in infection. In this experiment, the toilet was seeded to achieve target MNV concentrations near what has been reported in feces and vomit (*i.e.*, 9.5 × 10^9^ genomic copies / gram of feces, and 4.1 × 10^4^ genomic equivalents / mL of vomit)^28–30^. If the toilet water contains higher concentrations of virus due to GI illness, consequently, higher concentrations of aerosolized NV could be observed during toilet flushing.

Toilet flushing of NV contaminated waste could explain how two previous studies were able to detect aerosolized NV during WWTP^18,19^. Other studies have detected high concentrations of NV in wastewater, where the concentration varies across season^38–40^. Therefore, flushing waste contaminated with NV may not only be an aerosolization source but result in NV exposure among workers at WWTP. In addition, it has been suggested throughout the COVID-19 pandemic that aerosolization of SARS-CoV-2 via a flushing toilet may explain transmission in various locales^41,42^.

Previous studies have measured particle concentrations reported different sized particles, however, these studies only analyzed one location and height around the toilet^24,43^. The results of the study presented in this paper were similar to the results reported by two previous publications; with one study conducted at a toilet lab and the other study conducted in hospital bathrooms, both around FOM type toilets^24,43^.

A large proportion of particles generated from flushing the toilet were less than 1 µm, with 0.3 µm particles being the most abundant. The mean particle concentration was highest behind the toilet. This observation could have a significant impact where contaminated droplets could impact or settle onto surfaces behind the toilet and the toilet flush handle. Surfaces behind the toilet may be neglected during cleaning. Particle concentrations were higher at 0.15 and 0.25 m above the toilet than level with the toilet bowl. Knowlton et al. sampled at the same height as the toilet bowl, therefore that study could have underestimated total bioaerosol generated from flushing the toilet^24^. Particle concentrations at Heights 0.15 and 0.25 m above the toilet bowl were similar for all particle sizes. Due to the possibility that the bioaerosol created may travel to the breathing zone and result in inhalation, particles present above 0.25 m from toilet flushing should be investigated.

A limitation of this study was the inability to assess the viability of aerosolized MNV. Aerosol collection may have influenced MNV viability. Both the SKC BioSampler and Coriolis µ sampler are designed to maintain target organism viability using liquid impingement. Shearing forces produced by high flow rates have been suggested to be a reason for viability degradation^44^. However, no studies have directly investigated this phenomenon. In addition, it is possible that MNV remained viable, and some virus was reaerosolized during sampling. Lemieux et al. described the reaerosolization of bacteria can be influenced by liquid-based samplers and the sampler’s flowrate. Thus, viral particles suspended in reaerosolized droplets may have remained intact and viable. Therefore, further investigation into the viability of aerosolized MNV is needed^45^.

The bathroom used for sampling was a public bathroom that was inaccessible to the public during sampling periods. However, the ventilation, temperature, and relative humidity were set to typical building design specifications across all trials. Furthermore, sampling took place over a 30-min period and the airborne MNV concentrations reported represent an average over the sampling period. Virus concentrations likely are highest during the flushing event, therefore reported MNV concentrations are likely an underestimate. However, these data are impactful as they could inform bystander exposure for individuals entering the bathroom after contamination has occurred. Particle concentrations and bioaerosol could have been distributed differently due to ventilation changes during the study. Therefore, we averaged the particle concentrations for particle size across trials as an attempt to account for day-to-day variability.

## Conclusions

In conclusion, aerosolized MNV generated from flushing a FOM type toilet was detected. This is the first study to determine an indoor aerosolization source for MNV, a surrogate for NV and may be representative of other viral pathogens (*e.g*., rotavirus, SARS-CoV-2) aerosols generated during toilet flushing. This is the first study to evaluate particle concentrations at multiple positions and heights around a FOM type toilet. Particle concentrations suggest that an aerosol plume is created around the toilet, with higher average particle concentrations in the back of the toilet. The location of higher mean particle concentrations could have an impact on cleaning procedures, especially in healthcare facilities. Furthermore, the observations from this study suggests that virus (*e.g.,* NV) is aerosolized from toilet flushing and may contribute to human exposure.
